# Epigenetics of drought-induced trans-generational plasticity: consequences for range limit development

**DOI:** 10.1093/aobpla/plv146

**Published:** 2015-12-18

**Authors:** Jacob Alsdurf, Cynthia Anderson, David H. Siemens

**Affiliations:** 1 Integrative Genomics Program, Black Hills State University, Spearfish, SD 77799, USA; 2 Present address: Division of Biology, Kansas State University, Ackert Hall, Room 315, Manhattan, KS 66506-4901, USA

**Keywords:** *Boechera stricta*, DNA methylation, drought tolerance, epigenetic association analysis, glucosinolate chemical defence, MS-AFLP, range limits, trade-off

## Abstract

Genetic variation gives plants the potential to adapt to stressful environments that often exist beyond their geographic range limits. However, various genetic, physiological or developmental constraints might prevent the process of adaptation. Alternatively, environmentally induced epigenetic changes might sustain populations for several generations in stressful areas across range boundaries, but previous work on *Boechera stricta*, an upland mustard closely related to *Arabidopsis*, documented a drought-induced trans-generational plastic trade-off that could contribute to range limit development. Offspring of parents who were drought treated had higher drought tolerance, but lower levels of glucosinolate toxins. Both drought tolerance and defence are thought to be needed to expand the range to lower elevations. Here, we used methylation-sensitive amplified fragment length polymorphisms to determine whether environmentally induced DNA methylation and thus epigenetics could be a mechanism involved in the observed trans-generational plastic trade-off. We compared 110 offspring from the same self-fertilizing lineages whose parents were exposed to experimental drought stress treatments in the laboratory. Using three primer combinations, 643 polymorphic epi-loci were detected. Discriminant function analysis (DFA) on the amount of methylation detected resulted in significant combinations of epi-loci that distinguished the parent drought treatments in the offspring. Principal component (PC) and univariate association analyses also detected the significant differences, even after controlling for lineage, planting flat, developmental differences and multiple testing. Univariate tests also indicated significant associations between the amount of methylation and drought tolerance or glucosinolate toxin concentration. One epi-locus that was implicated in DFA, PC and univariate association analysis may be directly involved in the trade-off because increased methylation at this site on the genome decreased drought tolerance, but increased glucosinolate concentration.

## Abstract (precis)

Offspring phenotypes may be altered by environments that their parents lived in. These environmentally-induced trans-generational effects may be mediated by epigenetic mechanisms such as DNA methylation. Little is known about the role of such epigenetic effects in evolution; however, it is expected to facilitate evolution. To expand geographic range, it is thought that most species would have to adapt via evolution by natural selection to stressful environments beyond range boundaries. Contrary to expectations, we show that DNA methylation in an upland mustard species may underlie a drought-induced trans-generational tradeoff that may constrain the process of adaptation to stressful environments at lower elevations.

## Introduction

Understanding the factors and processes affecting species range limits is a fundamental goal in ecology and evolution, and a central concern for predicting the consequences of climate change (Parmesan *et al*. 2005; Gaston 2009; Sexton *et al*. 2009; Wiens 2011). Because most transplant experiments show poorer performance across range boundaries (Sexton *et al*. 2009 for review), many range margin populations must face stressful environments that they are not adapted to. Therefore, understanding what prevents this adaptation may be a key to understanding the development of range limits. Since there is often sufficient genetic variation within range margin populations, if there are also no barriers to dispersal, possible constraints on the process of adaptation include gene flow and trade-offs (Sexton *et al*. 2009). Although not much is known about adaptive quantitative genetic variation across ranges, studies of neutral genetic markers usually only show slight declines in genetic variation at range margins (Eckert *et al*. 2008). If range margin populations are also geographically and genetically isolated, a focus of studies on range limit development should be on molecular, physiological or developmental trade-offs (Kawecki 2008). At low latitudinal or altitudinal range limits, for example, populations are thought to more commonly face both abiotic and biotic stressors (Ettinger *et al*. 2011), the simultaneous response to which might result in conflicts or trade-offs (both ecological and evolutionary) possibly contributing to range limit development (Siemens *et al*. 2009).

Here, we studied the role of environmentally induced epigenetics (i.e. DNA methylation) in an apparent trade-off between abiotic and biotic stress responses that might influence altitudinal range limit development in the upland mustard (Brassicaceae) *Boechera stricta.* Mustard plants (Brassicaceae) include ∼3700 species, several crop species (cabbage, radish, canola, etc.) and the model for molecular plant biology, *Arabidopsis thaliana*. Despite this diversity, mustards generally inhabit high-altitude temperate regions where populations have patchy distributions. The altitudinal range of *B. stricta*, for example, is typically between 1700 and 3000 m (Song *et al*. 2006). At lower altitudinal range boundaries (1700 m), isolated *B. stricta* populations face drier conditions where attack by generalist insect herbivores and inter-specific competition may also increase (Siemens *et al*. 2009; Siemens and Haugen 2013). In a previous work (Alsdurf *et al*. 2013), a drought-induced trans-generational plastic trade-off was reported in *B. stricta* between drought stress tolerance and chemical defence allocation that could influence range limits. Offspring of parents who were drought treated had increased drought tolerance; however, they produced lower levels of glucosinolate (GS) toxins, which provide a chemical defence against generalist herbivores. This trade-off might contribute to low-elevation range limit development in *B. stricta* because it is likely that both drought tolerance and defence are needed to expand the range of *B. stricta* to lower elevations (Siemens *et al*. 2012; Siemens and Haugen 2013).

The molecular basis of epigenetic effects includes DNA methylation and chromatin remodeling (van Straalen and Roelofs 2012). DNA methylation occurs mainly on cytosine bases in CpG pairs, and is mediated by a small set of methyltransferases that methylate in different circumstances. Chromatin remodeling involves histone tails that may be methylated, ribosylated, ubiquitinylated, sumoylated, phosphorylated, acetylated and may interact with DNA methylation. Mechanisms of chromatin remodeling are complex and not fully understood. On the other hand, DNA methylation is more readily studied. To quantify methylation patterns, several methods are available such as methylation-sensitive restriction enzymes, which one can use for ‘epi-genotyping’, and bisulfate sequencing, which is used to identify methylated cites on a DNA sequence. Methylation-sensitive restriction enzymes, for example, have been used for population genomics to determine whether methylation was under selection (Herrera and Bazaga 2009), and in population genetic association studies (Herrera and Bazaga 2011).

DNA methylation is a mechanism of epigenetic transcriptional gene regulation that can be environmentally induced and inherited without changes to DNA sequence (Bird 2002; Akimoto *et al*. 2007; Henderson and Jacobsen 2007; Jablonka and Raz 2009; Verhoeven *et al*. 2010). Therefore, we hypothesized that DNA methylation could be involved in the previously documented trans-generational plastic trade-off between drought tolerance and chemical defence (Alsdurf *et al*. 2013). We predicted that patterns of DNA methylation would vary among offspring whose parents were differentially drought treated. Additionally, because such differential methylation among treatments should have a functional basis by affecting gene expression, we also predicted that we would find significant associations between variation in the functional traits and the epigenetic markers.

We used the methylation-sensitive amplified fragment length polymorphism (MS-AFLP) assay to elucidate differential patterns of DNA methylation in offspring whose parents were exposed to either control or drought watering treatments. The MS-AFLP uses isoschizomeric restriction enzymes, HpaII and MspI, that are sensitive to different forms of methylation (McClelland *et al*. 1994; Reyna-López *et al*. 1997). HpaII and MspI both digest un-methylated CCGG sites and cause differential cleavage depending on the pattern of methylation at this sequence.

## Methods

### Study organism

*Boechera stricta* is a genetically diverse, predominantly self-fertilizing perennial and close relative of *A. thaliana* that ranges across western North America at higher altitudes, typically 1700–3000 m (Song *et al*. 2006, 2009; Lee and Mitchell-Olds 2011). Unlike *Arabidopsis* in North America, *B. stricta* and many other species of *Boechera* are native, occur in natural habitats and, because of longer life cycles, face and presumably adapt to more ecological stressors (Mitchell-Olds 2001; Lovell 2011; Rushworth *et al*. 2011). Here, we focussed on a potential mechanism of trans-generational environmentally induced variation. However, there also exists significant quantitative genetic variation within and among populations of *B. stricta* (Siemens *et al*. 2009; Prasad *et al*. 2012). Genetic variation represented by five low altitudinal range margin populations in the Black Hills, South Dakota, USA, was used for greater inference, but this variation was controlled for by splitting the same sib-families into environmental control and treatment groups.

### Experimental design

Functional phenotypic data and tissue for DNA extraction were from a growth chamber experiment in which the plants in the offspring generation were differentially watered (control and drought) and whose parents had also been differentially watered, as described in Alsdurf *et al*. (2013). Briefly, in the parent generation, 384 plants representing 64 full-sib-families from five relatively low-elevation populations were exposed to three watering treatments (control watering during the basal rosette stage and throughout reproduction (CC), drought watering only in the basal rosette stage (DC) and drought watering through both stages (DD)). Drought treatments included less water and less often as monitored mainly by flat weights and growth rates, and ultimately correlated reproductive fitness effects. Offspring from 10 of the parental sib-families, each representing all three parental watering treatments, were used in the offspring experiment; therefore, genetic variation could be controlled in analyses to detect trans-generational plasticity. The 10 families also represented the population variation—2 families per population. To assess drought tolerance, the offspring were also exposed to experimental watering treatments, but only during the rosette stage.

In the offspring experiment, the 10 parental sib-families and the 3 parent drought treatments were represented within each of the 14 planting flats. Thus, there were 420 plants total (30 plants/flat × 14 flats). The watering treatments in the offspring generation were administered among flats (seven controls, seven drought treated). Here, we used 110 of the offspring plants: 33 with a parental history of control watering (CC), 39 with partial drought history (DC) and 38 with complete history of drought (DD). There were mainly two replicates in each watering treatment and sib-family combination (10 parental sib-families × 3 parental drought treatments × 2 offspring drought treatments × 2 replicates = 120 plants)—for some family-watering treatment combinations, there was only one replicate, hence the discrepancy between 110 and 120.

Several functional response variables were measured in the offspring generation to assess drought tolerance and defence and their associations with the trans-generational drought treatments (Alsdurf *et al*. 2013). Drought tolerance for each sib-family was measured as differential growth between watering treatments (Simms 2000), as carbon isotope ratio [δ^13^C, a measure of water use efficiency (WUE)] and as shoot dry weight. Defence was measured as the concentration of the three common GS toxins in the basal rosette leaves of *B. stricta*: 1-methylethyl, 2-hydroxy-1-methylethyl and 6-methylsulfinylhexyl GS. Leaf tissue for weights and extractions were conducted 9 weeks after drought treatments began (Alsdurf *et al*. 2013). The protocol and additional references for GS extraction, separation and quantification were given previously (Alsdurf *et al*. 2013). In the parent generation (Alsdurf *et al*. 2013), drought treatments reduced rosette size (40 %), flowering date (10 %) and fruit production (30 %), but not seed size or mass. Shoot size was positively correlated with reproductive output, and is also correlated with over-winter survivorship across the range boundary in the field (Siemens *et al*. 2009); therefore, plant size in this system can be used as an indicator of fitness and for evolutionary inferences. We were not able to measure reproduction in the offspring generation because tissues were used for GS, carbon isotope ratio and DNA methylation analyses. Instead, we relied on the above correlations of size and fitness (both reproduction and survivorship) for evolutionary inferences. However, interactive effects, such as costs of the trans-generational plasticity, might affect the relationship, but the existence of this cost has not been investigated.

### Methylation-sensitive amplified fragment length polymorphism

The MS-AFLP protocol used was based on Yu *et al*. (2011). DNA was extracted from leaf tissue using DNAeasy Plant Mini Kit (QIAGEN). Adaptors and primers are listed in Table 1. Two sets of restriction and ligation reactions were performed, one with HpaII and the other with MspI. The HpaII/EcoRI digestion started with 2 µL of 103 NEB buffer 2, 1 µL of HpaII (5 U), 15 µL of genome DNA (500 ng) and 2 µL of ddH_2_O were added into a 1.5-mL centrifuge tube. The same was done for the MspI/EcoRI digestion, except 1 µL of MspI (5 U) was used. The mixtures were then incubated at 37 °C for 2 h for complete digestion. The reaction was stopped by incubating at 65 °C for 20 min. Each of the reactions was continued with an EcoRI digestion. For EcoRI digestion, 3 µL of 103 EcoRI buffer, 1 µL of EcoRI (10 U), 20 µL of HpaII digestion system and 6 µL of ddH_2_O were mixed. Mixtures were incubated at 37 °C for 2 h, and then incubated at 65 °C for 20 min to stop the reaction. In the ligation reaction, 4 µL of 10× T4 DNA ligation buffer, 1 µL (5 pmol µL^−1^) of EcoRI adapter, 1 µL (50 pmol µL^−1^) of HpaII/MspI adapter, 30 mL of HpaII/EcoRI digestion product, 0.5 µL of T4 DNA ligase (40 U) and 2.9 mL of ddH_2_O were mixed. Reactions were incubated at 16 °C overnight. The reaction was subsequently stopped by incubating the mixture at 65 °C for 20 min.

**Table 1.** EcoRI/HpaII/MspI adapters and primers.

Primer/adapters	Abbreviation	Sequence (5′–3′)
EcoRI adapter		CTCGTAGACTGCGTACC AATTGGTACGCGTC
EcoRI primer	Primer E	GACTGCGTACCAATTC
Primer E + 2 (EcoRI selective primer)	Primer E1	Primer E-AA
	Primer E2	Primer E-AG
HpaII/MspI adapter		GACGATGAGTCTCGAT CGATCGAGACTCAT
HpaII/MspI primer	Primer HM	ATGAGTCTCGATCGG
Primer HM + 3 (HpaII/MspI selective primer)	Primer HM 1	Primer HM-AAT
	Primer HM 2	Primer HM-ATC
	Primer HM 3	Primer HM-TCC

Both HpaII and MspI products were subjected to pre-selective amplification with the following mixture: 3 µL of 5× Green GoTaq buffer, 1.5 µL of MgCl_2_, 0.3 µL of dNTPs, 1.5 µL of 10× bovine serum albumin (BSA), 0.1 µL of GoTaq polymerase, 1 µL of EcoRI primer (primer E) (50 ng µL^−1^), 1 µL of HpaII/MspI primer (primer HM) (50 ng µL^−1^), 1 µL of ligation product and 5.6 µL of ddH_2_O ultra-pure. These polymerase chain reactions (PCRs) were performed as follows: (i) 94 °C for 3 min; (ii) 20 cycles of 30 s denaturing at 94 °C, 1 min annealing at 60 °C and 1 min extension at 72 °C and (iii) 10 min at 72 °C for template extension. The presence of the fragments was checked using a 1.5 % agarose electrophoresis gel and 5 µL of pre-selective amplification product.

Selective amplification of the pre-selective products was carried out using three primer combinations. Those primer combinations were obtained by combining EcoRI primers E1 and E2 with the three HpaII/MspI primers HM1, 2 and 3 (Table 1). Both EcoRI and HpaII/MspI primers had two or three selective bases. The EcoRI and HpaII/MspI adapters and primers were synthesized by Integrated DNA Technologies, Coralville, IA, USA (www.idtdna.com) and mixed in the following quantities: 3 µL of 5× Green GoTaq Buffer, 1.5 µL of MgCl_2_, 0.3 µL of dNTPs, 1.5 µL of 10× BSA, 0.1 µL of GoTaq polymerase, 1 µL of EcoRI primer (primer Ex) (50 ng µL^−1^), 1 µL of HpaII/MspI primer (primer HMx) (50 ng µL^−1^), 1 µL of ligation product and 5.6 µL of ddH_2_O ultra-pure. Selective PCRs were performed as follows: (i) 94 °C for 5 min; (ii) 36 cycles of 30 s denaturing at 94 °C, 30 s annealing at 56–65 °C and 1.0–1.4 min extension at 72 °C and (iii) 10 min at 72 °C for template extension. Annealing was initiated at a temperature of 65 °C, which was then reduced by 0.7 °C for the next 12 cycles and maintained at 56 °C for the subsequent 23 cycles. The extension time was increased by 1 s for the last 24 cycles. The presence of fragments was checked using a 2 % agarose electrophoresis gel and 5 mL of selective amplification product.

### Methylation-sensitive amplified fragment length polymorphism marker scoring and error rate estimation

Autoanalysis detection of MS-AFLP fragments was done in GeneMapper v.4.1 (Applied Biosystems, Foster City, CA, USA) to minimize bias (Bonin *et al*. 2007). First, 12 MS-AFLP samples and their technical replicates were compared to determine the autoanalysis settings (Holland *et al*. 2008) of bin width = 1, peak height transmittance = 100 and minimum fragment length = 100, which was the recommended setting for autoanalysis and produced the lowest error rate when comparing replicates. Error rate estimation (Pompanon *et al*. 2005) among 30 technical replicates was the number of mismatches divided by twice the number of epi-loci.

### Epi-genotypes, coding and statistical analysis

For each of the three primer combinations (Table 1 and described in the previous section), the epi-genotypes were determined for each fragment size detected. Variation in the fragment patterns (presence/absence) at these fragment size epi-loci was caused by the differential cutting of the two restriction enzyme combinations EcoRI/HpaII and EcoRI/MspI. The fragment size epi-genotypes are referred to as fragment ‘conditions’ in Schulz *et al*. (2013) (Table 2). Condition I is the presence of fragments for both restriction enzyme combinations, indicating no methylation at the restriction site. Condition II occurs when a fragment is produced from MspI, but not from HpaII, indicating that internal cytosine is either fully or hemi-methylated at the restriction site. The opposite, when a fragment is produced by HpaII, but not MspI, is Condition III, indicating hemi-methylation of an external cytosine. Condition IV refers to the absence of fragments and indicates either full-methylation of external cytosine, full-methylation of both cytosines, hemi-methylation of either cytosine or possibly a mutation at the restriction site. Since we were comparing the same sib-families among drought treatment combinations, any variation from the drought treatments would not be caused by mutations at the restriction site.

**Table 2.** Restriction site methylation status inferred from isoschizomers HpaII and MspI sensitivities (‘+’ indicates enzyme cuts; ‘−’ enzyme does not cut), the condition labels used in Schulz *et al*. (2013) and the codings used here based on the average amount of methylation that could be inferred from the fragment patterns. Methylated cytosines are shown in grey.

	Methylation status	HpaII	MspI	Condition (Schulz *et al*. 2013)	Amount of methylation coded
CCGG GGCC	No methylation	+	+	I	0
**C**CGG GGCC	Hemi-methylation of external cytosine	+	(−)	III	1
C**C**GG GG**C**C	Full-methylation of internal cytosine	−	+	II	2
C**C**GG GGCC	Hemi-methylation of internal cytosine	−	+	II	
**C**CGG GGC**C**	Full-methylation of external cytosine	(−)	−	IV	3
**CC**GG GG**CC**	Full-methylation of both cytosine	−	−	IV	
**CC**GG GGCC	Hemi-methylation of both cytosine	−	−	IV	
Mutation	Unknown	−	−	IV	

For statistical analysis of MS-AFLP data, the conditions, or epi-genotypes as we also refer to them here, are first coded. We used multivariate statistics to analyse differences in methylation patterns among treatments. Analysis of molecular variance (AMOVA) requires binary data coded here as 0s or 1s (Excoffier *et al*. 1992); thus, MS-AFLP data are categorized as methylated (e.g. Conditions II and III) or un-methylated (e.g. Condition I). Because of the ambiguity of Condition IV in population genetic studies where genetic variation at the restriction site may occur, Condition IV data have been excluded from both methylated and un-methylated categories (Schulz *et al*. 2013). Here, we included Condition IV in the methylation category since the ambiguity caused by variation in restriction site nucleotide sequence does not exist when comparing ecological treatments using the same set of sib-families (i.e. the same restriction site nucleotide sequences were used across ecological treatments). Binary matrix data from GeneMapper v.4.1 were formatted according to package *msap* (Pérez-Figueroa 2013) and R function *MSAP_calc.r* (Schulz *et al*. 2013) specifications and imported into R statistical computing environment (R Core Team 2013) to score and quantify types of methylation resulting from MS-AFLP assay **[see** **Supporting Information—File S1****]**.

We also used an alternative coding scheme based on the amount of methylation at the restriction site, which allowed for alternative statistical analysis. When the conditions are ordered I, III, II and IV, the amount of methylation at the restriction site, on average, increases (Table 2). Thus, we used a rough quantitative coding 0, 1, 2 and 3 for the ordered conditions, respectively. This coding allows for alternative multivariate statistics, such as discriminant function analysis (DFA), that was originally designed by Fisher for quantitative data (Afifi and Clark 1984). To our knowledge, this coding and analysis have not yet been used on MS-AFLP data; yet, the amount of methylation may have functional effects. Discriminant function analysis determines whether there are certain combinations of the response variables (epigenetic loci in this case) that may be used to distinguish groups (e.g. the parental watering treatment groups in offspring generation). We conducted separate DFA for offspring control and drought treatments after determining that parent drought treatments in the offspring could not be distinguished in the total data set. We used classical backward stepwise DFA in SYSTAT13 (Systat Softwar, Inc., San Jose, CA, USA) with default *F*-value and tolerance settings. We only used polymorphic epi-loci in the analysis because the data set without the low-polymorphic epi-loci was more likely to satisfy assumptions of multivariate normality. However, DFA did not allow us to control for potentially confounding factors, such as planting flat, sib-family or development. As stated above under ‘Experimental design’, there were 110 plants total in the analysis among the three parental and two offspring generation watering treatments.

To control for these other confounding effects, we also constructed principal components (PCs) from the polymorphic epigenetic loci, and then asked whether the PCs varied among drought treatments, controlling for the confounding factors in an analysis of covariance (ANCOVA). If a PC varied significantly across drought treatments, we then examined PC component loadings for each MS-AFLP epigenetic locus. Epigenetic loci with large component loadings, either positive or negative, were examined for their associations with defence and drought tolerance measures to identify possible candidate genetic loci for trait regulation.

Thus, we also conducted univariate epigenetic association analyses using quantitative epigenetic codes (0, 1, 2 and 3 in Table 2), correcting for multiple loci testing (Laird and Lange 2011). However, we did not assume any mode of inheritance (i.e. dominance, recessive, additive or co-dominant) as is done in genetic association analyses. For each trait (shoot weight, carbon isotope ratio and GS concentration of the three common GS in *B. stricta*), we conducted a regression analysis against the coded MS-AFLP epi-genotype values, controlling for unmeasured random variation among planting flats, genetic variation among full-sib seed families and development (initial seedling size). For drought treatments, we conducted multinomial logistic regression, using coded epi-genotype values as the dependent response variable and drought treatment as the independent variable. We conducted separate logistic regression analysis within each drought treatment (control and drought) of the offspring generation to determine the effects of parent drought treatments. For each statistical test and epi-locus, we used the Simes (1986) experiment-wise α-rejection level, which is based on ordered *P*-values. We rejected the null hypothesis when *P*(*i*) < (*i*/*M*)*α*, where *M* was the total number of polymorphic epigenetic loci, or where *M* was the number of loci implicated in PC analysis (highest component loading values) or *M* was all loci used in the discriminant function. We used *α* = 0.1 to allow for all possible candidate epigenetic loci.

## Results

The methylation-sensitive AFLP analysis was conducted on 110 individual *B. stricta* plants, resulting in 235, 236 and 172 polymorphic epi-loci for the 3 primer combinations E1&2/HM1, E1&2/HM2 and E1&2/HM3, respectively (see Table 1 for primer abbreviations). But, when the epi-loci were coded for the amount of methylation (0, 1, 2 and 3—see Table 2), most of the variance (75 %) in the methylation was explained by just 50–100 of the epi-loci, depending on the primer combination **[see** **Supporting Information—Fig. S1****]**. The error rates for the primer combinations varied from 3 to 9 %, which was within the 2–10 % error rate range usually found in AFLP studies (Avolio *et al*. 2011; Price *et al*. 2012).

### Multivariate analyses

Differences in DNA methylation in the offspring generation caused by parent drought treatments were more apparent under offspring control watering conditions compared with drought, but this result depended on the type of methylation data and analysis used. While the AMOVA did not distinguish the parental drought treatments in the offspring, the DFA did. For the analysis of binary MS-AFLP data (0 = un-methylated Condition I, 1 = methylated Conditions II, III and IV), differences were detected among all combinations of parental and offspring watering treatments (AMOVA: Phi_ST = 0.03735, *P* = 0.0026, Table 3), but these differences in methylation were caused by drought treatments during the offspring generation. Separate AMOVAs for offspring control and drought treatment groups showed no differences between parental drought treatments, even when each primer combination was analysed separately (*P*s > 0.05).

**Table 3.** Analysis of molecular variance on offspring MS-AFLP data generated from all three primer combinations. Groups are all combinations of parental (CC, DC and DD) and offspring (C and D) watering treatments.

	df	SSD	MSD	Variance	Phi_ST	*P*-value
Among groups	5	448.7	89.73	2.023	0.03835	0.0026
Within groups	106	5526	52.13	52.13		
Total	111	5974	53.82			

**Figure 1.**
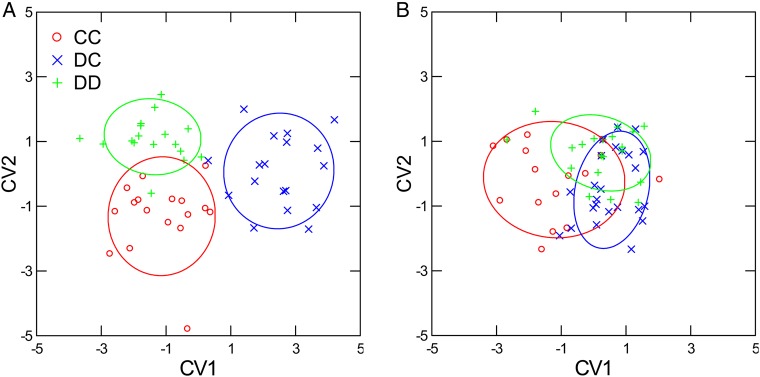
Methylation-sensitive amplified fragment length polymorphism CV (canonical variable) bi-plots. Separate DFA was conducted for offspring control (A) and drought (B) watering treatments. Canonical variables were constructed from backward stepwise DFA. Data are individual offspring plants. Different colours represent parent drought treatments (CC = controls, DC = drought treated only during vegetative stage and DD = drought treated during vegetative and reproductive stages). Also shown are 68 % confidence circles for each parent drought treatment.

In contrast, using DFA on the quantitative methylation data, there were significant differences among parent drought treatments in separate analyses for each of the different primer combinations (e.g. primer combination 1, Fig. 1). These analyses only included the 50 most polymorphic epi-loci for each primer combination and were conducted separately within each offspring drought treatment. For example, under offspring control watering conditions, 43.2 % of the most polymorphic epigenetic loci from primer combination 1 were included in the final discriminant functions (*P*s < 0.05) that distinguished group centroids (Fig. 1A: *F*_38, 64_ = 3.373, *P* < 0.001), whereas under offspring drought conditions, only 8 % of epigenetic loci were included in the discriminant functions, although the centroids were again still distinguishable (Fig. 1B: *F*_10, 110_ = 2.705, *P* = 0.001). Discriminant function analysis could not distinguish among parent drought treatments without separate analysis for offspring control and drought treatments (*F*_88, 144_ = 0.894, *P* = 0.714). Similar results were detected for the other primer combinations when offspring drought treatments were analysed separately (parent watering treatment group centroids distinguished in offspring: *P*s < 0.05).

**Figure 2.**
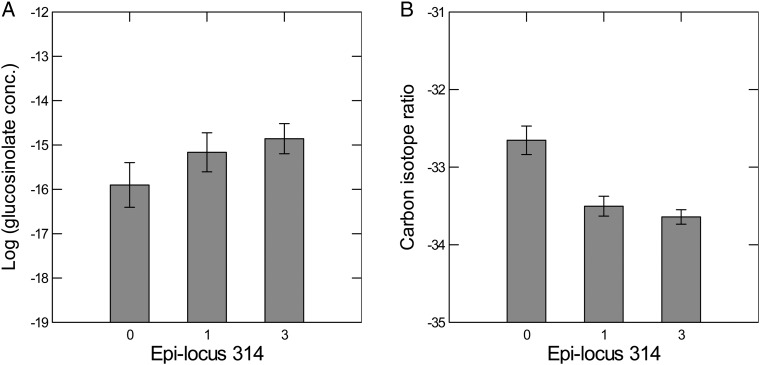
Methylation profile of epigenetic locus 314. 1-Methylethyl GS levels (A) and carbon isotope ratios (B) across different MS-AFLP types (coded 0, 1 and 3) at locus 314. There were too few code 2s at locus 314 for analysis. Epi-locus 314 is a site on the genome that is a candidate area for the co-regulation of defence and drought tolerance.

For primer combination 1 that produced the most polymorphic epigenetic loci **[see** **Supporting Information—Fig. S1****]**, the first five PCs, constructed from the quantitative codes (0. 1, 2 and 3 in Table 2), each explained at least 1/50 (1/number of epi-loci tested) or 2.0 % of the variance in MS-AFLP polymorphisms and were therefore considered for further analyses (Afifi and Clark 1984). But only PC5 varied significantly across parent drought treatments (Table 4). The epigenetic locus that had the highest loading coefficient (*r* = −0.658) on PC5, epigenetic locus 314, is a candidate for the co-regulation of defence and drought tolerance as predicted by the trade-off (Fig. 2). Epigenetic locus 314 was also included in CV1 and CV2 in DFA (see above), and also implicated in univariate analyses (see below).

**Table 4.** *F*-ratios from ANCOVA for the effects of parent drought treatment on MS-AFLP PCs generated from primer combination 1. The PCs were constructed using the quantitative coding values for offspring plants grown under control watering conditions. Significant multivariate test statistic (Wilks's *λ*) protected subsequent univariate tests from Type I errors. *P ≤ 0.05; **P ≤ 0.01; ***P ≤ 0.001.

	df	PC1	PC2	PC3	PC4	PC5	Wilks's *λ*
Parent treatment	2	0.250	0.119	0.019	0.285	7.411**	2.075*
Flat	6	10.963***	6.165***	5.041***	1.043	5.115***	7.500***
Family	6	1.054	0.646	0.638	0.468	1.548	0.704
Seedling size	1	2.556	0.522	0.220	0.243	0.983	0.978
Error	33

### Univariate association analyses

Univariate analyses also indicated significant associations between MS-AFLP polymorphisms and drought treatments or trait values (Table 5). Of the 50 most polymorphic epigenetic loci from primer combination 1 tested, 47.7 % were implicated. Most detectable were associations with offspring drought treatments or associations with δ^13^C (WUE). Also, epi-locus 314 was again found to be significant and a candidate for co-regulation of defence and drought tolerance. Although the univariate analysis for epigenetic locus 314 did not implicate GSs, we found significant associations between PC5 and GS level (regression analysis: 2-hydroxy-1-methylethyl GS, *t* = 2.050, *P* < 0.05, and 1-methylethyl GS, *t* = 2.880, *P* < 0.01). Recall that PC5 varied significantly among parental drought treatments (Table 4) and its highest component loading was for epigenetic locus 314.

**Table 5.** Univariate association analysis. Significant associations with epigenetic loci produced from primer combination 1. We used *α* = 0.1. *P*-values are in parentheses beside the test statistic. Glucosinolate level (GS) and carbon isotope ratio (δ^13^C) abbreviated. ^1^Tests conducted from samples in offspring drought treatment only. Null hypothesis for each test *i* rejected when *P*(*i*) < (*i*/*M*)*α*, where *M* was the total number of polymorphic epigenetic loci (Simes 1986).

Locus	Shoot weight	Offspring drought treatment	Parent drought treatment	δ^13^C	GS
	*F*	*χ*^2^	*χ*^2^	*F*	*F*
13	6.966 (0.0003)	22.712 (0.00005)		3.606 (0.024)	
34	5.990 (0.001)	41.042 (6 × 10^−9^)			
33	5.575 (0.001)	19.199 (0.0002)			
9		17.713 (0.001)			
23		11.145 (0.011)		4.552 (0.005)	
39		10.895 (0.012)		4.346 (0.007)	
26		15.521 (0.001)		3.213 (0.027)	
332	14.014 (0.006)	14.014 (0.003)			
346		16.895 (0.001)			
264		15.912 (0.001)			4.7375^1^ (0.009)
326		12.643 (0.005)			
192		11.793 (0.008)			
223		10.988 (0.012)			
133		12.301 (0.024)	14.599 (0.006)		
202				4.833 (0.004)	
314				4.785 (0.004)	
373				4.311 (0.007)	
259				3.854 (0.012)	
230				3.633 (0.016)	
124			15.499^1^ (0.017)		
344			20.285^1^ (0.002)		
df	3, 96	3	6	3, 96	3, 28

## Discussion

Little is known about the role of epigenetics in range limit dynamics. For range margin populations at their physiological limits, adaptive stress-induced trans-generational plasticity (Herman and Sultan 2011) might allow for immediate range expansion. And the effect could last many generations (e.g. Herman *et al*. 2012), eventually becoming permanent in some cases (Jablonka and Raz 2009; Cortijo *et al*. 2014). However, this scenario ignores the possibility of epigenetic constraints, and it fails to acknowledge the importance of documenting epigenetic mechanisms directly.

The mechanisms of trans-generational plasticity include epigenetic and other, maternally inherited factors. The other, maternal factors include, in the case of plants, differential seed provisioning with mineral nutrients, proteins, carbohydrates or lipids that may affect seed mass, and other seed mass-independent factors including hormones, mRNA, small RNA or secondary metabolites (Richards *et al*. 2010; Herman and Sultan 2011). Epigenetic mechanisms include DNA methylation (attachment of a methyl group to cytosine in DNA) and several kinds of histone modifications (e.g. acetylation or methylation of histone protein) that affect gene expression (Richards 2006, 2011; Herman and Sultan 2011; Holeski *et al*. 2012; Kovalchuk and Kovalchuk 2012). Epigenetic effects are more likely to persist for several generations, especially in plants where chromatin effects such as DNA methylation are less often erased at meiosis, possibly because of the modular and diffuse nature of soma–germ line interface (Richards 2006). Epigenetic effects, therefore, may be more likely to play a role in range limit dynamics. To evaluate the role of epigenetics in the trans-generational effects studied, we checked for correlated DNA methylation.

Here, we examined patterns of DNA methylation in a previously documented drought-induced trans-generational plastic trade-off that could possibly limit range expansion (Alsdurf *et al*. 2013). Evidence for the involvement of epigenetics in this trans-generational drought-induced constraint was from (i) differences in patterns of DNA methylation among offspring from different parent drought treatments (Fig. 1) and (ii) significant associations in offspring between MS-AFLP loci and tolerance or defence trait variation (Table 5). In particular, we were able to identify an example of a methylated site on the genome that may be involved in the co-regulation of defence and drought tolerance traits (Fig. 2). At this site, the patterns of variation in the amount of methylation were associated with defence and drought tolerance traits in opposite ways. In this way, DNA methylation could be used to identify candidate genes or regulatory sequences involved in such trade-offs. However, in our case, we were not able to isolate the DNA fragment on a gel, and therefore, we could not sequence and identify the sequence that the fragment originated from. Other methods for studying DNA methylation, such as bisulfate sequencing, would be more useful for identifying such methylated DNA regulatory sequences.

Population genetic association analysis (Foulkes 2009) is very different in principal than the epigenetic association analysis conducted here. Genetic association analysis involves naturally occurring polymorphisms and is conducted on large samples of unrelated individuals. Therefore, it is based on linkage disequilibrium (LD) and also called LD mapping. For success, LD mapping usually requires many markers. Ideally, mapped single nucleotide polymorphisms (SNPs) occurring throughout the genome are used with high-throughput SNP chips in what is known as genome-wide association analysis. However, other molecular markers such as AFLPs reveal relatively high amounts of genomic variation in non-model species and therefore can also be used for genetic association analysis with much effort. If present, any population sub-structuring (i.e. stratification, admixture or inbreeding) must be controlled for in association mapping because of confounding effects on LD that can lead to false positives (Laird and Lange 2011). In contrast, the MS-AFLP association analysis that we conducted was used to associate phenotypes and epigenetic markers generated by environmental treatments. Because DNA methylation affects gene expression, we assumed that most of the epigenetic loci that we detected were within genes or their regulatory regions. As such, far fewer epigenetic markers are required for successful association analysis. To control for confounding effects of genetic variation within treatments, we used sib-families as covariates in the epigenetic association analysis.

We suggest that DNA methylation of key genes in the abscisic acid and jasmonic acid/ethylene signalling pathways, such as transcription factors that are known to be involved in the crosstalk between these pathways (Fujita *et al*. 2006), may be involved in the trans-generational trade-off. Of course, further study into the nucleotide sequences found associated with the epigenetic candidate loci is needed to reveal gene promoter or regulatory regions involved.

It should be noted that most of the univariate epigenetic association analyses between traits and methylation involved drought tolerance and not defensive traits (Table 5). This may be because we only included drought treatments that may only affect defence signalling and induction indirectly. Because of the complexity of the experiment, i.e. multiple generations, sib-families, drought treatments, flats and development, we did not also include herbivore-induction treatments that may elicit adaptive trans-generational defence responses (Holeski *et al*. 2012) and may also have resulted in more significant associations involving defence.

Other association studies using MS-AFLP markers have been conducted to understand the extent of the variation and heritability of epigenetic marks within and among natural populations. In natural populations of the violet *Viola cazorlensis*, epigenetic variation using MS-AFLP markers was associated with long-term patterns of herbivory (Herrera and Bazaga 2011). Variation in DNA methylation was also found in natural populations of the perennial herb *Helleborus foetidus*, and these patterns persisted at least across male gametogenesis (Herrera *et al*. 2013). In contrast, our study was experimental and, therefore, more directly implicates the associations of environmental factors, epigenetic marks and trait values. But similar to the other studies, we used a non-model organism and we also documented genetic variation in the patterns of DNA methylation. That is, there was often a significant sib-family effect in the univariate statistical analyses. However, we controlled for genetic variation in DNA methylation by using the same sib-families in all environmental treatment combinations. There were enough sib-families and replicates within families to prevent effects from any variation in methylation within families.

There are few other studies on drought and epigenetics, even in model organisms (but see Wang *et al*. 2010), probably because it is difficult to quantify empirical drought treatments. Instead, other studies on epigenetics and abiotic or biotic stress have focussed on other more quantifiable environmental variables and used model organisms. For example, nitrogen-deficiency stress in rice (Kou *et al*. 2011), pathogen induction in tobacco (Boyko *et al*. 2007), salt stress in maze (Tan 2010) and cold stress in maze (Shan *et al*. 2013). These are a few examples of the current work involving cytosine methylation influencing gene expression and its heritability. We focussed on drought because it was reported to be a relevant ecological gradient across *B. stricta* low-elevation range boundaries (Siemens *et al*. 2009). In general, stress in plants has been shown to increase methylation levels for non-stress-adapted plants and decrease methylation levels in stress-adapted plants (Choi and Sano 2007; Kou *et al*. 2011; Gao *et al*. 2014).

## Conclusions

In conclusion, one might expect for adaptive trans-generational plasticity to facilitate range expansion; however, we show that there may be epigenetic constraints inhibiting this process. We suggest that such constraints may be caused by methylation of genes of major pleiotropic effects, such as transcription factors regulating drought tolerance and defence signalling pathways. But much more work is needed to understand the genetic basis of such trade-offs and in general the role of epigenetics in range dynamics.

## Data Accessibility

The full data set of all coded epi-loci for all primer pairs, treatments and phenotypes will be made available upon publication.

## Sources of Funding

Research reported was partially supported by the National Institute of General Medical Sciences of the National Institutes of Health under award number 8 P20 GM103443-12. The content is solely the responsibility of the author and does not necessarily represent the official views of the National Institutes of Health. J.A. was partially supported by NSF-STEM grant DUE 0728553 and the BHSU Integrative Genomics graduate program.

## Contributions by the Authors

J.A. and C.A. performed MS-AFLPs; both J.A. and D.H.S. conducted statistical analyses and wrote the manuscript. All authors helped interpret results and commented on the manuscript.

## Conflict of Interest Statement

None declared.

## Supporting Information

The following additional information is available in the online version of this article –

**File S1.** R Script.

**Figure S1.** Distribution of variances among the quantitative epigenetic codes (0, 1, 2 and 3—see Table 2) for each MS-AFLP primer combination (see Table 1).

Additional Information
