# Supplementary material for: Epigenetics of drought-induced trans-generational plasticity: consequences for range limit development
Source: AoB Plants. 2015 Dec 18;8:plv146. doi: 10.1093/aobpla/plv146 (PMC4722181; doi:10.1093/aobpla/plv146)
Supplement: Additional Information [file supp_8_plv146_index.html]

Epigenetics of drought-induced trans-generational plasticity: consequences for range limit development — Additional Information 

# Epigenetics of drought-induced trans-generational plasticity: consequences for range limit development

## Additional Information

Additional Information

- Supplementary File 1 - docx file
- Supplementary File 2 - docx file
