# Supplementary material for: Epigenetics of drought-induced trans-generational plasticity: consequences for range limit development
Source: AoB Plants. 2015 Dec 18;8:plv146. doi: 10.1093/aobpla/plv146 (PMC4722181; doi:10.1093/aobpla/plv146)
Supplement: Additional Information [file supp_plv146_plv146supp_file1.docx]

**R Script**

R Script 1. *MSAP_calc* script

>source("*MSAP*_calc.r")

>d<-Extract_MS-AFLP_epigenotypes ("histP1.txt","Mix2", "MS-AFLP_Mix2.txt",1,TRUE)

>p<-descriptive_parameters ("MS-AFLP_Mix2.txt","MS-AFLP_Mix2_descr.txt")

(Note: *R script 1 is the MSAP_calc* script, which just helped identify the different conditions indicated in Table 2. We also visually inspected the data from each epigenetic locus to make sure that the condition designation was correct.)

R Script 2. *Msap*

>library(msap)

>msap("histC.csv",name="histC", loci.per.primer=c(235,236,172))

(Note: R script 2 is the *msap script*, which was used to generate the simple unmethylated/methylated scoring 0 and 1 used in AMOVA. According to the Perez-Figueroa ([2013](#_ENREF_37)) paper, the msap package generates the “Herrera scheme” scoring explained in the Schulz et al. (2013) paper.)
