# Supplementary material for: Epigenetics of drought-induced trans-generational plasticity: consequences for range limit development
Source: AoB Plants. 2015 Dec 18;8:plv146. doi: 10.1093/aobpla/plv146 (PMC4722181; doi:10.1093/aobpla/plv146)

**File 2.** Figure. Distribution of variances among the quantitative epigenetic codes (0, 1, 2 and 3 – see Table 2) for each MS-AFLP primer combination (see Table 1).


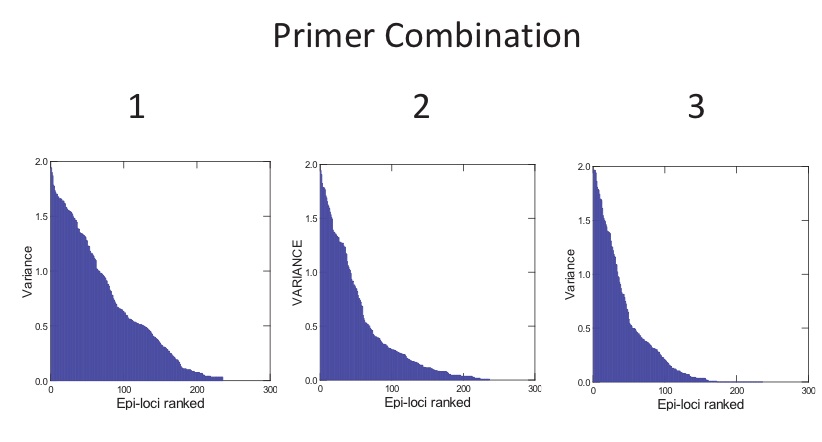

Supplement: Additional Information [file supp_plv146_plv146supp_file2.docx]
